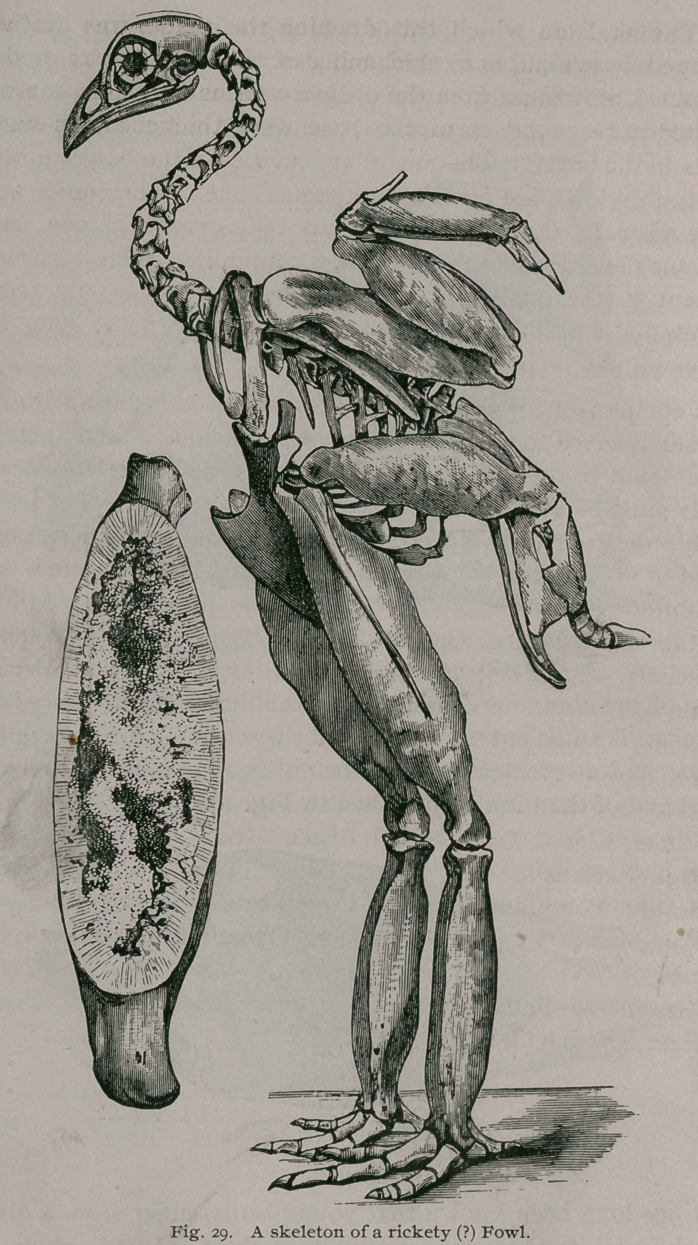# Rickets in Monkeys, Lions, Bears and Birds

**Published:** 1889-01

**Authors:** J. Bland Sutton

**Affiliations:** Hunterian Professor Royal College of Surgeons, England; Assistant Surgeon Middlesex Hospital, London


### THE JOURNAL

— OF —

### COMPARATIVE MEDICINE AND SURGERY.

VOL. X. JANUARY, 1888. No. 1

### ORIGINAL COMMUNICATIONS.

### ART. I.—RICKETS IN MONKEYS, LIONS, BEARS AND BIRDS.

BY J. BLAND SUTTON, F. R. C. S.,

*Hunterian Professor Royal College of Surgeons, England; Assistant Surgeon Middlesex Hospital, London.*

In the year 1650 a small book was published in London, entitled "A Treatise of the Rickets; Being a Disease Common to Children. Published in Latin by Francis Glisson, George Bate, and Ahasuerus Regmorter, Doctors in Physick and Fellows of the College of Physitians at London." Five years before the appearance of Glisson's book, Dr. Whistler, then President of the College of Physicians, London, printed a short treatise on rickets (1645), this being the first book published on the subject. Dr. Norman Moore, in his interesting essay, *The History of the First Treatise on Rickets*, states the name rickets was in popular use thirty years or more before the appearance of Glisson's book, and notwithstanding the fact that Whistler's work appeared five years before Glisson's, the credit of the elucidation and first complete description of the disease is due to Glisson.

Since the date of Glisson's book (which passed into a third edition), rickets has been ably investigated clinically, pathologically and experimentally by very many observers, and a long list of monographs, dissertations, lectures, and papers by distinguished authors has been devoted to the elucidation of its pathology and the best means of treatment. Therefore, it seems a little strange more attention has not been devoted to this malady in animals. It has long been known that domestic animals and wild beasts in captivity suffer from rickets; this is attested by the fact that the Museum of the Royal College of Surgeons, London, possesses specimens exemplifying the condition of the skeleton when affected by rickets in lions, cats, and monkeys, several of the preparations being marked "Hunterian."

Many observers[1] have also incidentally noted the occurrence of rickets in monkeys, porcupines, deer, and the like, but so far as I am aware no one has attempted to deal systematically with the disease as it occurs in animals. In the following pages an attempt will be made to describe in some detail the chief features of this very remarkable disease as it occurs in monkeys, lions and bears. The bulk of the material has come under observation during my attendance at the Prosector's room of the Zoological Society, London, during the last seven years.

### RICKETS IN MONKEYS.

This disease is extremely frequent in monkeys living in captivity in London. Nearly half the total number of young monkeys introduced into the Zoological Society's Garden die rickety, provided they live a few months after reaching London. The changes in the skeleton develop so rapidly that a Capuchin monkey apparently in good health, and thriving well when introduced into the cages, died horribly deformed by rickety changes in four months.

The *clinical* features of the disease are the following: Instead of leaping from rail to rail, fighting, or otherwise displaying evidence of agility, the monkey grovels on the floor, and, if pushed, shrieks as though the movement caused pain. By degrees the power of locomotion becomes considerably impaired, the creature displaying signs of paralysis in the lower limbs, finally ending in paraplegia, accompanied with priapism, incontinence of urine and

1. Chief amongst these are Otto, Hunter, Sandifort, Crisp, Murie and Percy.

fæces. The mode of progression is now characteristic. The hinder portion of the body being paralysed, the monkey is obliged to use the long fore limbs as crutches, and swing the trunk between them. At this stage deformity of the chest is obvious. A well marked kyphotic curve of the spine is established in severe cases, and the creature presents a very pitiable condition. A sudden change in the weather, or exposure to a draught of cold air, sets up a slight attack of bronchitis, and as sure as a rickety monkey develops a cough, be it even a slight one, the animal soon succumbs.

On examining the dead body, attention is chiefly arrested by the kyphotic curve of the spine and the "pot belly." If the monkey is very rickety the thorax will be so soft that it yields to the pressure of the fingers like a soft leather boot. The sternum is thrust forward and bent upon itself, the convexity of the curve being directed backwards. In many cases the distance between the xiphoid cartilage and the symphysis pubis is seriously diminished. The limb bones may, or may not be curved, in many cases they are abnormally thickened throughout their entire length, whilst in others the ends alone are enlarged.

Fig. 1. Transverse section of the thorax of a rickety Monkey. H. Heart. L,L. Lungs. T. Trachea. Œ. Œsophagus. A. Aorta. P. Pericardium. (Proc. Zool. Soc.)

Before proceeding to discuss in detail the changes as they affect the bones individually it may be useful to consider some of the effects these skeletal deformities exert upon the contained viscera. These may be ascertained by freezing the monkey and then sectioning it in any direction desired. A transverse section through the middle of the thorax of a very rickety monkey is represented in Fig. 1. Here we see the lungs represented as two narrow bands, flattened by the yielding of the softened ribs to atmospheric pressure, the trachea is pushed to one side, and the œsophagus is in close contact with the spine; the heart is pushed forward and closely pressed by the thoracic walls, instead of hanging freely in its pericardium between the two pleural bags. When the thoracic viscera are hampered in this way, it is not surprising that even a slight attack of bronchitis should prove fatal to a rickety monkey. But this pressure on the heart leads to other ill-effects. When conducting dissections of rickety monkeys, my attention has often been arrested by a curious appearance presented by the right ventricle; in many cases the wall is so exceedingly thin that the blood in the ventricular cavity can be seen through it, giving to the tissue a peculiar purple tint, exactly like that of a venous nævus.

Fig. 2 Sagittal Section through thorax of Rickety Monkey. Showing "flexion" of the heart.

The only way to account for this attenuation of the ventricular wall was to regard it as due to pressure. A rickety monkey was frozen and bisected sagittally, as in Fig. 2, it was then clearly seen that in consequence of the kyphosis the sternum had become bent upon itself, and the "knuckling" thus produced squeezed the heart between the sternum and the spine; as a result of this continued pressure, atrophy of the ventricular wall resulted. It has been already mentioned that when the kyphosis is extreme the xiphoid and symphysis tend to meet, and the anterior aspect of the pelvis looks upwards. Fig. 3.

This leads to a diminution of the capacity of the abdomen and consequent crowding of the viscera. The monkey thus becomes "pot-bellied," the diaphragm encroaches upon the sternum, and the sternum in the long run bends upon itself. Thus, not only is the thoracic cavity narrowed laterally, but also from below, thus encumbering the main respiratory and circulatory organs—lungs and heart. In addition to the kyphotic curve there are often two short, but well marked, lateral curves in the anterior part of the thoracic region of the spine.

Fig. 3. The thorax, spine and pelvis of a rickety monkey. The anterior aspect of the pelvis has come to be directed upwards in consequence of the kyphotic curve of the spine.

Serious changes also occur in the pelvis, for the ossa innominata, softened by the disease, yield to the weight of the body, which they transmit to the heads of the femora, so that the transverse diameter is seriously narrowed and in severe cases the opposite sides of the true pelvis come together, nipping the vagina and urethra between them. This leads to two conditions. In the case of the urethra, obstruction to the flow of urine may end in hypertrophy of the muscular tissue of the bladder. In females, the uterus and its appendages are pushed upwards into the cavity of the false pelvis, Fig. 4, and being there at the mercy of the intestines, the uterus often becomes acutely flexed, either backwards or laterally, sometimes, though rarely, it is bent forwards.

We must now direct attention to the spine. It has long been known that paraplegia is extremely common in monkeys living in confinement in this country, and for a long time no satisfactory explanation was forthcoming. In 1875[1] Paul Gervais published an interesting article, entitled "*De l'hyperostose chez l'homme et les animaux.*" Among the specimens there figured is a vertebra from an animal named *Pachyacanthus*, dug up near Vienna. It is a singular specimen, and shows a condition which is very rarely met with, viz., general obliteration of the spinal canal, due to overgrowth of bone. This supplied the hint, and I divided the spinal column in all rickety monkeys. This is what was found:—The general overgrowth and softening of bone so common throughout the skeleton had not spared the vertebræ with their various processes, but they had enlarged and encroached upon the spinal canal, and thus exercised general slow compression upon the spinal cord. When the creature stood, the pressure of the superincumbent weight would cause the vertebral bodies to bulge and compress still more the spinal cord and the nerves as they emerge from the various intervertebral foramina. I am not aware of any recorded cases of such general narrowing of the neural canal; and it is easy to explain why it has been overlooked, for it is usual to expose the cord by removal of the vertebral arches, thus destroying the relative size of the cord to the spinal canal; whereas, if a transverse section of the column be made with the cord *in situ*, the change is obvious. The cord and nerves, when examined microscopically, exhibit all the changes found in the grey and white matter when the cord has been compressed from other causes, such as cancer, tumor, vertebral caries, etc. A vertebra of *Pachyacanthus*, showing its narrowed canal, is shown in Fig. 5, and a section through a vertebra, showing the compressed cord *in situ* from a rickety monkey, in Fig. 6. When we come to consider the carnivora, other causes of paraplegia in rickety monkeys will be described.

Fig. 4. Rickety pelvis of Monkey, showing the mode of displacement of uterus and its subsequent retroflexion.

---

1 *Journal de Zoologie*, 1875, Vol. iv, p. 272.

We must now consider the anatomy of the the changes produced in the skeleton of monkeys by rickets. Before doing so it may be stated that, in the human subject, rickets is, in the major-

Fig. 5. Vertebra of Pachyacanthus, showing the narrowed spinal canal. (After Gervais, l. c.)

ity of cases, a curable affection. Up to the present time I have never seen a case of arrested or cured rickets in a monkey.

Fig. 6. Transverse Section of the Vertebral Column, with the Cord *in situ*, to show the mode by which the cord is compressed by overgrowth of the surrounding bone. From a monkey.

The gross morbid anatomy of the rickety skeleton may now be considered, commencing with the skull. The bones composing a monkey's skull, like those in the skulls of other mammals, fall into two categories—those preformed in membrane and those preceded by cartilage. Rickets, as a rule, attacks these two varieties of bone in a very different manner. The membrane-bones in the early stages soften and thicken slightly, whilst the cartilage-bones become thin and also somewhat softened. Not infrequently I have divided the skull of a rickety monkey with a stout knife as easily as if it were gutta percha.

Fig. 7. Under surface of the skull of a rickety monkey, showing the perforated cerebellar fossæ.

The cerebellar fossæ are occasionally perforated in very rickety monkeys (Fig. 7), and not infrequently the skull of a rickety monkey comes to hand with the bones of the vault measuring an inch in thickness, and those of the base thin and almost as transparent as parchment. Such thick skulls are most common in baboons, but they occur in other monkeys, and a very thick skull from a macaque is represented in Fig. 8. The specimen is preserved in the Museum of the Royal College of Surgeons. This thickening is not confined to the membrane bones of the cranial vault, but attacks also those of the face, so that, in severe cases, the skulls of monkeys resemble the famous Jadelot cranium.

Fig. 8. The Skull of a Rickety Macaque Monkey seen in horizontal section. It shows the thickened membrane bones.

It will be of interest to consider this remarkable skull somewhat in detail, for it has a good deal in common with the skulls of some rickety bears which will be described.

About 1745 or 1750 a skull was dug up from a depth of fifteen feet in the soil at the village of Sacy, near Rheims. This skull was presented by a physician of that town to Bernard de Jussieu. In 1799 Jadelot* published an account of the specimen, which was remarkable for its great thickness. The skull has been re-described by Paul Gervais.[1] The most important features presented by this skull are the following: The dentition shows it to have belonged to a child, aged about five years. All the bones of the cranium and face have acquired an extraordinary thickness; some of

---

**Description anatomique d'une tete humane extraordinaire, Paris, 1799.*

1 *Journal de Zoologie*, Vol. iv., p. 272. *De l' hyperostose chez l'homme et chez les animaux.*

the sutures have suffered obliteration, and the zygomatic arch is enormously thickened. Its various cavities, such as the antra, nasal fossæ and orbits are much contracted by the bony overgrowth; the lachrymal canal, and the various nerve-foramina in the basis cranii are contracted, and some of them, the optic foramina and the perforations in the cribriform plate are obliterated. The walls of the skull vary from an inch to an inch

Fig. 9. The Jadelot Cranium.

and a quarter in thickness, and the overgrowth of the maxillæ, including the alveolar ridges, is very great. The chief features of this skull are well shown in Figs. 9, 10, 10 a and 11.

Even a superficial study of the figures of this skull is sufficient to convince one that the patient to whom it belonged must have lost more or less the sense of smell, of sight, and probably that of hearing. This also differs from most rickety skulls by the fact that the cartilage-bones of the base as well as the membrane-bones of the face and cranial vault are thickened. That the

Fig. 10. The Base of the Jadelot Cranium.

changes in the Jadelot cranium are due to rickets I have never entertained any doubt, but it has served to perplex me, inasmuch as I did not, for several years, come across a skull among rickety mammals at all similar to this cranium. During the present year, however (1888), two young Assyrian bears were killed in the Zoological Gardens, London, on account of extreme paraplegia. An examination of the skeleton showed them to be the seat of severe rickety changes. The skulls were greatly thickened, the bones of the vault measuring three-quarters of an inch in thickness, yet they were so soft that the skull and vertebral column, including the vertebral centra, were cleft longitudinally by means of a stout

Fig. 10 a.

The mandible of the Jadelot Cranium.

knife. The paraplegia was due in part to the spongoid tissue developed between the epiphysial plates and the vertebral centra, as described in detail on page 13; but in one of the bears the arch of the atlas was much thickened, thus narrowing the diameter of the canal in this part of the column considerably. In addition to being par-

Fig. 11. The interior of the Jadelot Cranium (after Gervais.)

aplegic, one of the bears was totally blind; this was not due to cataract, as is so often the case with bears, nor to any gross aberration that could be detected in the globe; on carefully tracing out the optic nerve, it soon became clear that the overgrowth of bone had narrowed and almost obliterated the optic foramina, thus satisfactorily explaining the blindness. Many of the other nerves in the basis cranii are similarly nipped. Thus we have in the skull of this bear a condition of things analogous to the Jadelot cranium. The rickety nature of the bear's skull is borne out by the long bones of the limbs, which present all the usual features of rickets. I also succeeded in obtaining some sections across the optic foramen with the nerve *in situ* for the microscope.

The changes in shape of the thorax have been already considered in some detail. Some mention, however, must be made of the changes in the ribs. The most conspicuous is that known as "pigeon breast," produced by the softened ribs yielding to atmospheric pressure, whereby the transverse diameter of the thoracic cavity is seriously narrowed and the antero-posterior measurement lengthened. In severe cases this diameter may be greatly narrowed, in consequence of the bending of the sternum described on page 4. Large beads form at the junctions of the ribs and costal cartilages,[1] the enlargement being confined to the rib, and is most prominent on its pleural aspect. Often double rows of rickety beads exist on each side. The second row is situated between the costal cartilages and the angle of the ribs. As a rule, this second row of beads is limited to the ribs between the second and ninth. The beads in the middle of the rib-shafts are due to infractions, that is, the ribs are so bent in at the anterior portion of the chest that the shafts near the angles are fractured, but the periosteum remains intact, at least in the majority of the cases. The movement of the broken bones in respiration causes a large amount of thickening, due to provisional callus, leading to the bulgings in question. In the most exaggerated example of rickets which came under my notice (a Macaque monkey), seventy-six infractions were counted on the ribs alone.

We must now deal with the appendicular skeleton, commencing with the fore-limb:

*The Clavicle.* This bone is, as a rule, thickened, especially at the sternal end. Occasionally at the point of origin of the clavicular portion of the sterno-mastoid muscle the bone is found considerably bent, due, in all probability, to the action of this muscle in forced respiration. When it occurs, the deformity is usually symmetrical.

*The Scapula.* The most notable features in this bone is great thickening and extreme softening, so that the serratus magnus is able to produce "lipping" of the vertebral border.

---

1 Beading at the junction of the ribs and costal cartilages is explained by regarding the costal cartilage as of the nature of an unossified epiphysis belonging to the rib.

*The Humerus, Radius and Ulna.* The shafts of these bones become increased in their transverse diameters ; the periosteum is thick and succulent, and the epiphyses are often twice or thrice the normal size, whilst a red, vascular marrow fills the medullary cavity. The most distinctive signs are to be found at the epiphysial junctions, where, replacing the thin narrow streak of hyaline cartilage, a band of tissue is seen, frequently five, and in some cases ten, millimetres in thickness, the increased breadth being due to the bluish translucent tissue named by Guerin "spongoid-tissue." The lower epiphysis of the radius and the head of the humerus are the seats of the greatest change in the upper limb. The carpal and metacarpal bones share in the general disturbance. The long bones, in addition to the changes above described, undergo abnormal curving in the direction of their length, but these softened bones rarely break.

Fig. 12. Sagittal section of the Skull, Atlas and Axis of a rickety Bear. There is a cartilage islet in the epiphysis between the odontoid process and body of the axis.

*The Os innominatum.* The changes these bones undergo has already been referred to, so far as the shape of the pelvis is concerned. The only additional one that needs be mentioned is the inversion of the iliac crest due to the action of the abdominal muscles.

*The Femur, Tibia and Fibula.* With the exception of the changes connected with the pelvis, the description of the upper limb would apply to the bones of the lower extremities, viz.: thickened periosteum, curved shafts, enlarged epiphyses, irregular growing lines, softened texture, and increased vascularity. The changes are most obvious in the lower ends of the femur and tibia. Test: If rickets be suspected, an examination of the lower epiphy-

Fig. 13. Section of a rickety epiphysis. Femur of a baboon. a, a, a, cartilage-islets. (Path. Trans.)

Fig. 14. Section of a rickety epiphysis of the femur of a boy. There is a large cartilage-islet in the diaphysis.

sis of the femur will either confirm or disprove the suspicion, and in this way it serves as a ready test of the existence of rickety changes, as well as serving to indicate its severity or otherwise. A typical section of a rickety femur is shown in Fig. 13, and beside it is a representation of a similar section through the condyles of a rickety human femur from a child four years old. Fig. 14. These sections show a feature of some interest, in Fig. 13, indicated by the letters *a*, *a*, *a*, are some small patches of hyaline cartilage which have remained untransformed in the ossification of the epiphysis; these are known as "cartilage-islets," and a good sized one is represented in Fig. 14 in the diaphysis.

The cartilage-islets are of very common occurrence in rickets, and they are regarded as probably the source of some forms of enchondromata.

The association of rickets and enchondromata has been pointed out by Johannes Müller, Virchow, Lennoir, Lebert and others. Although cartilage-islets of the size shown in Fig. 14 are not common, nevertheless, in microscopic sections of rickety epiphyses they can nearly always be detected. Sometimes these locked-in pieces of cartilage grow abnormally and produce an enlargement of the surrounding bone, resembling a tumour. Such a condition is represented in Fig. 15. The parts

Fig. 15. The Scapula, coracoid process and upper extremity of the femur of a rickety monkey, showing enlargements due to locked-in pieces of cartilage. (Proc. Zool. Soc.)

were taken from a Macaque monkey. Each scapula presented a large rounded mass at the angle, a similar enlargement surrounded the base of the coracoid process, and the great trochanter presented itself as a large exostosis. The enlargements were symmetrical.

*Histology.*—Briefly, the changes met with in rickety bones may be summed up thus:—they are an exaggeration of the processes normally concerned in the formation and growth of bone. As bones increase in length at the epiphysial cartilages, and in width or thickness by a deposition of matter from the deeper layers of the periosteum investing the diaphysis, these tissues are the ones chiefly concerned.

On making a vertical section through a healthy long bone (the femur is the best and most convenient bone to select) taken from a young animal, we find at either end a certain portion of the shaft segmented off by a very thin but distinct and regular line of hyaline cartilage, usually one millimetre in thickness. If the tissue at this spot be submitted to the microscope it will be found that this apparently homogeneous line of cartilage presents *two* very distinct zones. The one farthest from the bone shaft exhibits the characteristic appearance of normal hyaline cartilage, the second, in immediate proximity to the growing bone, displays

Fig. 16. The microscopic appearances of a section through the lower end of the femur of a rickety monkey. (Semi-diagrammatic.) (Trans. Path. Soc.)

cartilage cells arranged in tiers of about six, eight, or ten, each column being separated from its fellow by a spiculum of calcareous matter. This arrangement extends the whole breadth of the epiphysial cartilage, so that in section these cellular columns are arranged as regularly as a phalanx of soldiers, and present a characteristic picture.

In rickets all this is exaggerated; a section through the epiphysis shows the line of cartilage to be several millimetres thick, instead of one; between this and the bone is a layer of variable thickness, formed of a peculiar gelatinous tissue known as the "spongoid tissue" of Guérin, possessing a bluish, translucent appearance. Examination with a microscope shows *three* zones, viz. :

1. A layer of normal hyaline cartilage exceeding many times its healthy thickness.
2. The layer of cells arranged in vertical columns, but consisting not of ten or twelve superimposed cells, but in severe cases of rickets as many as fifty or sixty may be counted in a single column.
3. Beyond these, a layer of irregular calcareous trabeculæ enclosing here and there "islets of spongoid tissue," and tracts of hyaline cartilage.

It is the proliferation of the cells in the second layer which gives rise to "spongoid tissue." It was noted, in dealing with the morbid anatomy of this disease, that the ends of the long bones become very much enlarged; the microscopic structure affords a satisfactory explanation. If a normal centre of ossification be carefully traced, it will be found to extend from a definite spot, known as the ossific centre. The border of advancing ossification is well marked, and the cartilage clearly and regularly disappearing. Such an epiphysis as this should be termed a "discrete epiphysis." In rickets things go on in a very disorderly way; instead of the earthy matter being deposited in a definite spot it is sprinkled irregularly throughout the terminal cartilage, so that it is no uncommon thing to find thirty or forty separate nuclei for such an epiphysis as that for the condyles of the femur. As these various nuclei become confluent, here and there little tracts of hyaline cartilage become enclosed by calcareous matter, constituting what I have termed "cartilage-islets;" these are often of sufficient size as to need no artificial help in detecting them. For such a condition as this the term "diffuse epiphysis" seems applicable, as implying a condition of things in which lime salts are deposited in disorder in a softened cartilaginous matrix.

Thus far observation has been limited to the ends of bones and the epiphysial cartilages, but changes no less important may be detected in connection with the periosteum. In the normal condition, the deeper layers of this tissue are adding to the bulk of the shaft by the deposition of fibrous lamellæ, which subsequently ossify.

In rickets the periosteum is very thick and succulent, the trabeculæ of fibrillated tissue pass into the bone shaft but do not undergo ossification, so that in a transverse section of the shaft of a long bone, the familiar concentric lamellæ of the Haversian system is replaced by a narrow band of osseous tissue in the midst of a fibrillated matrix containing no calcareous matter.

*Anomalous Forms of Rickets.*—In the vast majority of cases the rickety changes are manifested by all the bones of the skeleton, but in rare instances the disease may localize itself to one particular part of the skeleton, or may restrict itself to a few bones. These anomalous forms of rickets were rather a puzzle to me for some time, but now I have no doubt of their nature. In the baboons and also occasionally in the Macaques, a cranium will come to hand with the membrane bones of the vault enormously thickened, and yet no rickety changes can be detected in the other

Fig. 17. Sagittal section of a skull of a baboon, with the membrane-bones thickened by rickets.

bones of the skeleton, except, perhaps, some sponginess of the facial bones (Fig. 17.) In other cases the facial bones are equally affected with those of the vault. Were it not that a precisely similar thickening accompanies general rickety changes, it would leave us in doubt as to the nature of these thickened skulls. In the microscopic characters such bone presents a characteristic condition ; instead of an outer compact and an inner vitreous table separated by diploe, we have bones homogenous throughout. When recent, they may be cut with a knife like gutta-percha ; when dry, they have a porous look, like old morter. This appearance is due to the fact that when these bones are fresh, a large quantity of osteogenetic tissue enters into their composition; when this soft tissue is removed, by allowing the soft material to decay by maceration, a porous condition is left, this residue consisting chiefly of lime salts and imperfectly organized osseous matter.

Up to the present time I have not found a skull presenting that hard, almost ivory-like, density which characterizes the thickened skull in a case of cured rickets in the human subject.

There is good reason to believe that rickets may remain restricted to a few bones of the limbs. Again and again I have examined monkeys in which, perhaps, the humeri or the femora alone were thickened, and in one case both humeri and both femora were greatly enlarged, yet so soft, that they could be split longitudinally with a knife. Such a specimen is shown in Fig. 18.

Fig. 18. Anomalous rickets. The femur entire A. Longitudinally bisected B. Transversely divided C. The natural outline of the shaft is sharply indicated in B and C.

The parts are drawn of natural size. The normal femur is sharply indicated in the centre of the specimens, but near the ends of the bone it shades off indefinitely. The epiphysial lines however exhibit the changes peculiar to rickets, and the soft material in which the diaphysis is embedded, presents the structural changes seen in rickety bones.

This specimen is of interest, because it throws some light on many enlarged and thickened bones seen in museums, obviously taken from young animals, and presenting a dry, mortar-like appearance. On several occasions I have taken bones resembling in all respects the femur (Fig. 18), and, having thoroughly macerated them, allowed the bones to dry. The deseased femora treated in this way, resemble in every respect the dry porous bones from young animals so often labelled "osteoporosis" in our museums. The museums of St. George's Hospital, London, contains a skeleton of a rickety monkey, but it is labelled "a monkey's skeleton affected with general caries" (!)

### Rickets in Carnivora.

This disease, though not so common among carnivora as among quadrumana, is, nevertheless, extremely frequent among the larger members of the cat family, especially lions born in London. It is also a very fatal disease in lions, and gives rise to changes of great interest. As a rule, when this disease occcurs early, the limbs are most affected; if it supervenes at a later date, then the axial skeleton suffers most, and in some cases the changes are confined to the skull.

The changes in the bones, and at the epiphysial lines, are precisely similar to those seen in monkeys macroscopically and microscopically. In its effects upon the thorax the same changes arise in the ribs, viz.: beading at the line of junction of the rib with its costal cartilage, and in severe cases, a second line of beads near the angles of the ribs due to infractions. These appearances are well shown in Fig. 19, which represent one-half of the rickety thorax of a Binturong. Nevertheless, the severe deformity of the chest seen in monkeys rarely occurs in carnivora. On the other hand, we shall have to study some severe effects of the disease from which monkeys escape.

When the axial skeleton is grossly affected, one of the most interesting clinical symptoms is paraplegia. This arises in different ways, and in monkeys has a different cause to that which pertains in many carnivora, especially the Felidæ.

Anatomy teaches us that in mammals the final act in the development of a vertebra is the fusion of two complemental discs of bone with the centrum; these discs are familiar to us as the epiphysial plates. When these plates fuse with the centrum the the mammal is said to be adult, and further growth is arrested in this part of the skeleton.

Fig. 19. One-half of the thorax of a Binturong, showing the beading on the ribs.

As far as my knowledge extends, the discs are more rudimentary in man than in other mammals above the Prototheria (Monotremata); indeed, in him they are thin menisci. In Ungulates, especially the horse, they are very large; they reach their maximum in whales; in some of the larger species an epiphysial plate from a thoracic vertebra would, if fitted with legs, serve as a very useful stool. Among the cats these discs are relatively large, especially in lions. Before these plates fuse with the centrum they are separated by a layer of growing tissue, like the epiphysis of a long bone, and in rickety mammals this epiphysial line is liable to develop the same blue translucent tissue so characteristic of the disease. This has enabled me to demonstrate that in some cases at least, the paraplegia is due to the formation of an unusual amount of this tissue, which, sprouting from the epiphysial lines, projects into the canal, compresses the spinal cord, and leads to paraplegia. (Fig 20.) In the human subject paraplegia from this cause never occurs, and the explanation lies in the circumstance that the epiphysis of the human vertebræ are very insignificant when compared with those of lions and the majority of mammals.

Further inquiry brought to light several facts of interest. In some of my paraplegic and rickety cubs the condition of the vertebræ was not sufficient to explain the paralysis, and in some, the paraplegia was so general as to suggest that the medulla oblongata was the seat of compression. That this view has been fully confirmed will appear from what follows.

Fig. 20. Portion of the spinal column of a rickety Sloth Bear, shown in section to show the mode of compression of the cord. (Nat. Sise.)

We have already seen that when rickets affects the skull, the bones preformed in membrane are those most thickend by the disease. The lion, like most carnivorous mammals, has an ossified tentorium cerebelli. This, even in the largest lion is very little thicker than ordinary writing paper. When the skull vault becomes thickened in consequence of rickets, the enlargement extends to the tentorium. (Fig. 21.)

In such cases the enlarged tentorium resembles an exostosis, and it is easy to understand that this mass of bone pressing upon the cerebellum and medulla is sufficient to produce the various symptoms observed in such cases. The clinical and pathological features, however, differ with the age of the feline patients. We will first consider the matter in a cub.

In 1887 three lion cubs were born in the Gardens. At the age of two months paralytic symptoms were observed in one of them. The affected cub, instead of playing with its companions, preferred to remain quiet, became very thin, and grew slowly. When attempting to walk it advanced a few paces, then staggered, and finally the hind quarters rolled over. At the same time the head would rotate laterally, the eyes oscillate, and convulsions sometimes occurred. When in the sitting posture, so characteristic of cats, rhythmical movements of the head were at times detected. At the age of three months the cub died. Its head was frozen and bisected sagittally ; the following interesting condition was found :

Fig. 21. Sagittal section of a lion's skull affected with rickets. The tentorium is very large.—(Trans. Path. Soc.)

The tentorium cerebelli was abnormally thick, and presented at its anterior edge a rounded margin. This overgrowth of bone had pressed upon the vermiform process of the cerebellum, thus occluding the anterior part of the fourth cerebral ventricle, and preventing a free flow of fluid from the remaining cavities. As a consequence the lateral ventricles became greatly dilated, and the foramen of Monro, instead of being represented as a slit of the dimensions of a crowquill, was a wide oval aperture. The third ventricle was likewise dilated, and the infundibulum, instead of being a narrow tube ending in the pituitary body, became widely dilated, and formed part of the general cavity of the third ventricle. Fig. 22.

The bones of the skull vault are thicker than is usual in lion cubs at this age. The skeleton generally presented rickety changes. The *post-mortem* appearances fully explain the symptoms, which resemble those seen in children when the interventricular communications are obstructed by either tumours, inflammatory troubles or congenital defects.

Fig. 22. A sagittal section of the head of a lion cub, showing overgrowth of the tentorium and dilation of the ventricles—T, Tentorium; V, Lateral ventricle; I, Infundibulum. (Pro. Zoo. Soc.)

In lions which become affected with rickets about the age of six months, or the attack beginning early, is not severe; the lion may often survive a year or two. In such cases the effects upon the brain are somewhat modified, the only constant symptom being slow progressive paraplegia. A typical case occurred in a lion two-and-a-half years old: for many months it was paraplegic; ulcers appeared on the hind limbs, the tail become gangrenous, and for weeks there was incontinence of fæces and urine.

Fig. 23. Sagittal section of the cranium of a rickety lion, showing an enlarged tentorium, compressing the cerebellum and occluding the fourth ventricle.

On bisecting the head, the usually rickety changes were found and an abnormally thick tentorium. (Fig. 23.) This enlarged tentorium had compressed the cerebellum and obliterated the fourth ventricle. The Sylvian aqueduct and third ventricle are dilated: the lateral ventricles retained their normal size. In the cub we saw that the lateral ventricles were most affected. This variation is probably due to the fact that in the cub the yielding sutures of the cranium allowed the bones of the vault to yield to the intra-cranial pressure exerted by the accumulating cerebro-spinal fluid, whereas in the older lions the firm sutures withstand the pressure, hence the brain tissue suffers most. The changes were well illustrated in the following case: The skull-vault (Fig. 24), was taken from a rickety lion, two years old, born in the Gardens of the Zoological Society, London. It died after presenting severe paraplegic symptoms, such as have been already described. On removing the vault of the cranium, the tentorium was found to be greatly thickened, resembling an exostosis.

Fig. 24. Under view of the skull-vault of a rickety lion, with abnormal thickness of the ossific tentorium. V. F. The vermiform fossa. (One-fourth the natural size.)

The cerebellum was severely compressed, its vermiform process had not only obliterated the fourth ventricle, but had by its pressure produced a depression in its floor. (Fig. 25.)

It may here be mentioned that some rickety cubs, which early manifested signs of rickets, were promptly fed on bone-dust and cod-liver oil, made a good recovery, and were alive and active, presenting no signs of paralysis two years afterwards.

Fig. 25. A section of the cerebellum and medulla from the same case as Fig. 24, showing obliteration of the fourth ventricle.

With regard to the lion's born in London, there remains this remarkable peculiarity, that in spite of every care in feeding and keeping them in comfortable dens, they develop rickets, yet in Dublin, Manchester, and some other British towns, lions can be reared successfully in captivity.

*The Teeth.*—It has been known that in rickets the teeth are late in being erupted; this was attributed by McShaw[1] to diminution of the jaws and the large size of the teeth, but the cause, I believe, depends upon the abnormal thickness of the follicles. The teeth sacs furnish the cementum of a tooth and part of the surrounding loose ossific alveolar tissue. If the roots of the teeth be examined by dissecting away the walls of the maxillæ in a severe case of rickets, they will often be found embedded in a sac of considerable thickness, and unerupted teeth will be found thoroughly surrounded by a thick fibrous envelope, which, when examined microscopically, will be found made up of concentric laminæ of fibrous tissue with lamellæ of bone interspersed. That the tooth-sacs should enlarge in this way is not surprising, when we remember that rickets is especially prone to attack those membranes especially concerned in the production of bone. But there is an interesting condition of teeth for which rickets must be held responsible, viz.: some forms of odontones. In 1887 I drew attention to the relation of certain forms of fibrous tumours connected with the roots of teeth, especially in goats, and attributed various forms of odontones to different degrees of development and ossification of these fibrous capsules. In several of the specimens dissected by me, these odontones were symmetrical, and in some cases four tumours were present, one in each upper and one in each lower jaw. Thus rickets may be held responsible for some forms of odontones, but for fuller details the reader may consult the reference given below. [2]

---

1 *Med. Chir. Trans.*, Vols. xvii., and xxvi.

2 Trans. Odont. Soc. Gt. Britain, Nov. 1887.

The bear from which this drawing was made, was brother to the one whose skull is figured and described on page 13, and as I explained, it differed from the ordinary forms of rickets, inasmuch as the cartilage bones of the base were thickened, as well as

Fig. 26. Portion of the facial skeleton of a rickety bear, showing an enlarged tooth follicle. F.

those of membranous origin. An examination of the long bones, however, is sufficient to show that these changes are rachitic in nature, and a representation of the enlarged epiphysial line at the distal end of the ulna is furnished in Fig. 27.

Fig. 27. Rickety epiphysial line of the ulna of an Assyrian bear.

### Birds.

It has long been known that young birds suffer from a disease, which in all its characters resembles rickets. I have studied the disease chiefly in the rhea, emu, ostrich and pigeon. The most remarkable specimens of rickets known to me are two articulated

Fig. 29. A skeleton of a rickety (?) Fowl.

and complete skeletons of fowls preserved in the Museum of the Royal College of Surgeons. One of them is represented in Fig. 29. It is thus described in the last edition of the pathological catalogue :

"The skeleton of a fowl, of which the extremities are much enlarged by symmetrical thickening of the shafts of most of the bones. The humerus, radius, ulna and metacarpal bones are affected in the upper extremity; the femur, tibia, and metatarsal bones in the lower. The medullary cavities in the bones, normally empty in a fowl, are filled with porous bone. The joints appear to be normal. The disease consists of a growth of new bone of variable density, and in many places containing much fatty matter. Except in the quality of the bone produced, it would seem to correspond with other cases of general osteitis of the long bones either in man or the lower animals."

A comparison of the macerated bones of this bird with those already referred to under the head of anomalous rickets, will leave little doubt in the mind that the changes in the skeleton of these fowls must be attributed to rickets.

In order to make this account of rickets as complete as possible a list of vertebrates is appended, in whom I have detected unmistakable rickety changes in the skeleton.

Man, Chimpanzee, Orang, Gibbon, Monkeys of all kinds, but especially *Macaques*, *Baboons*, *Capuchins*, *Squirrel Monkeys*, Lemurs, Potto.

Lions, Tigers, Hyæna, Coati, Cynictis. Bears—*Assyrian*, *Sloth*, *Brown*.

Cats, Dogs, Binturong, Raccoon, Raccoon-like dog, Seals.

Camel, Llama, Deer, Horse, Sheep, Goats.

Pigs, Babirussa.

Beaver, Porcupine, Rabbits, Coypu rats, Spermophilus.

Kangaroos, VulpinePhalangers, Opossums, Dasyures (especially.)

Lizards especially Monitors.

Emu, Ostrich, Rhea, Pigeons.

## Figures and Tables

**Fig. 1. f1:**
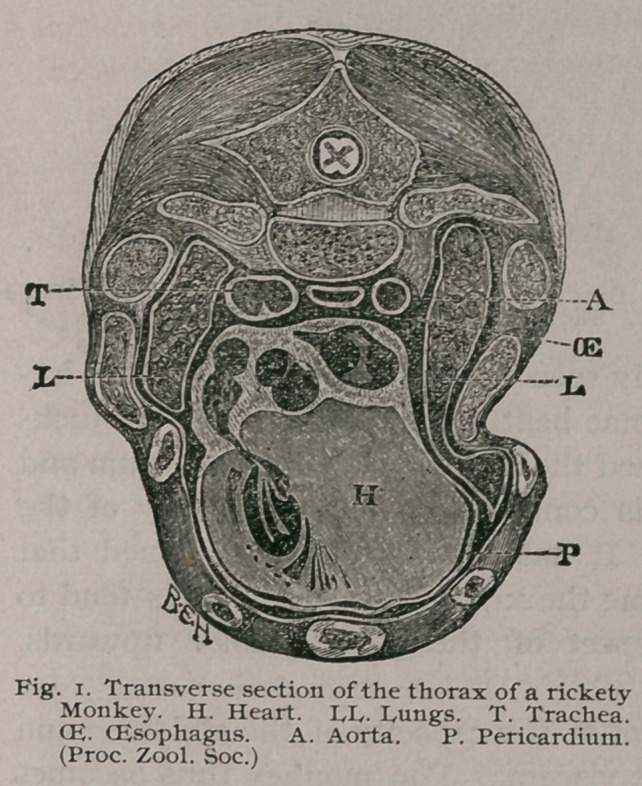


**Fig. 2 f2:**
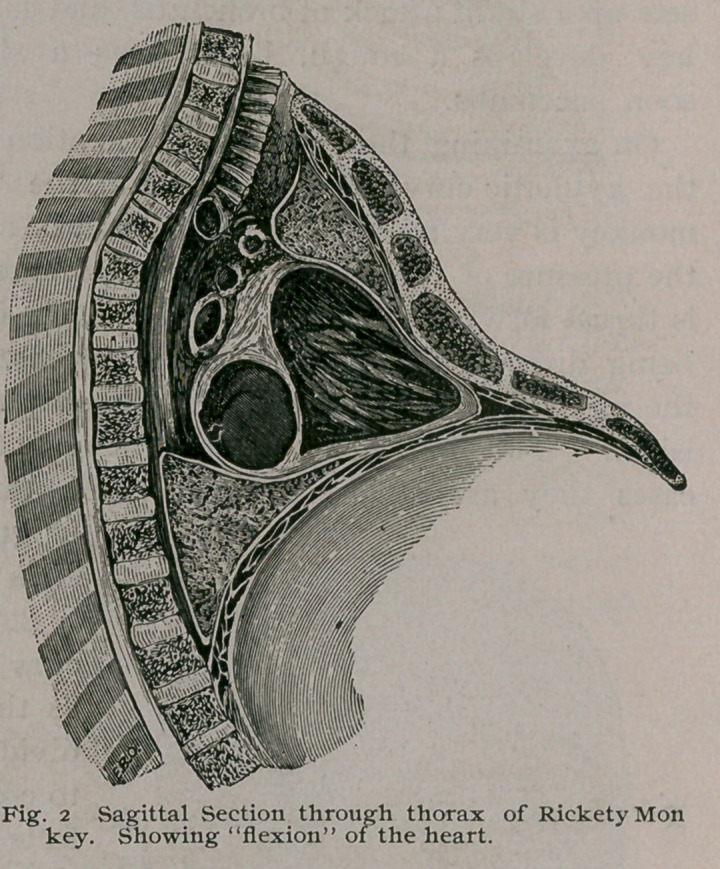


**Fig. 3. f3:**
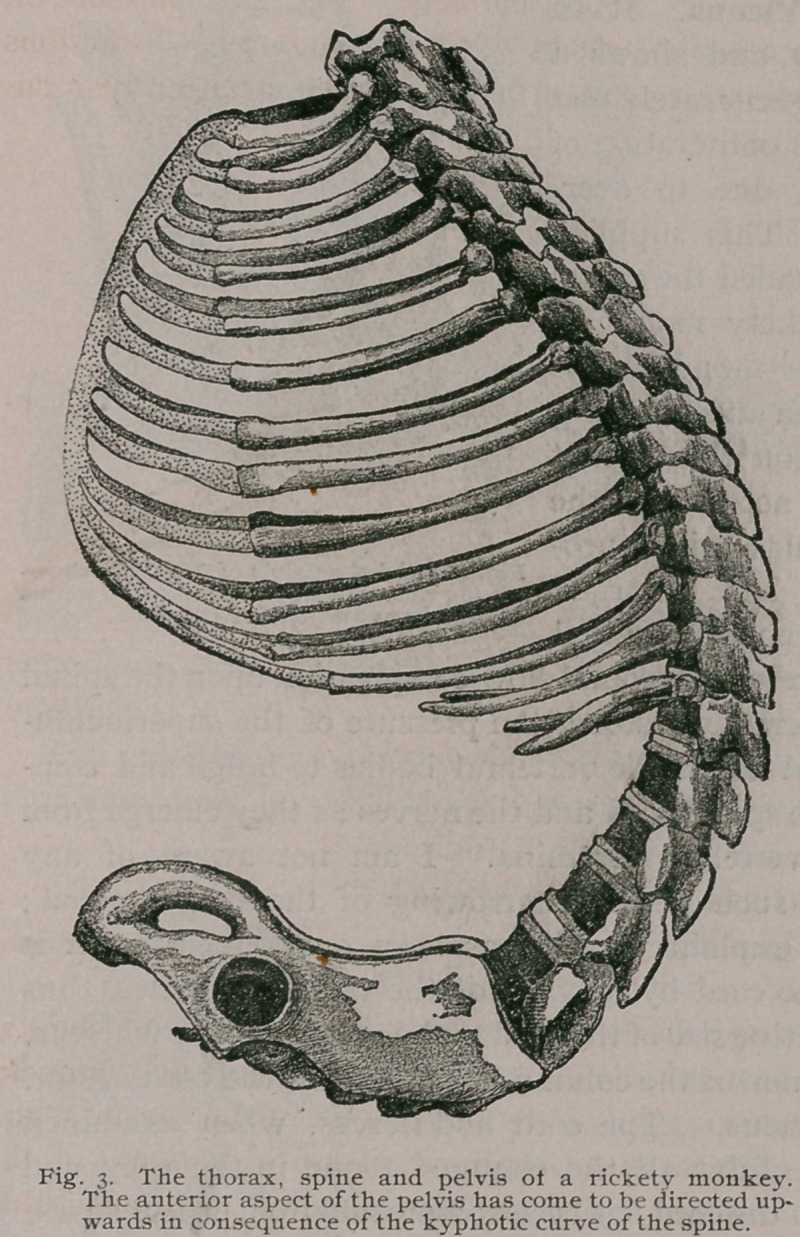


**Fig. 4. f4:**
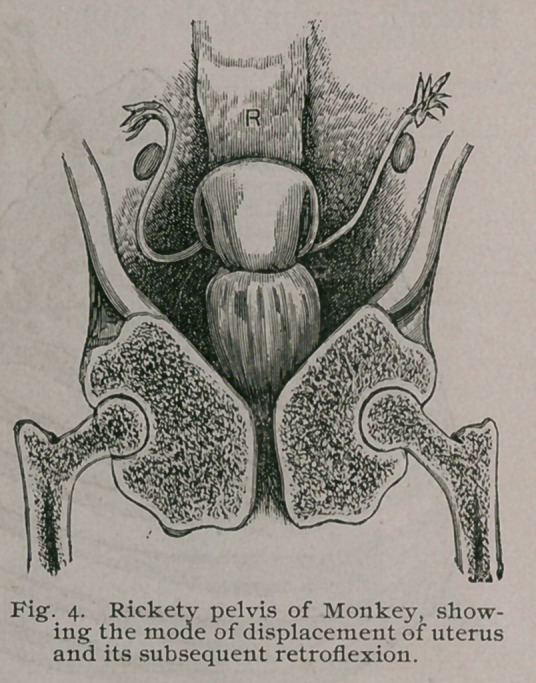


**Fig. 5. f5:**
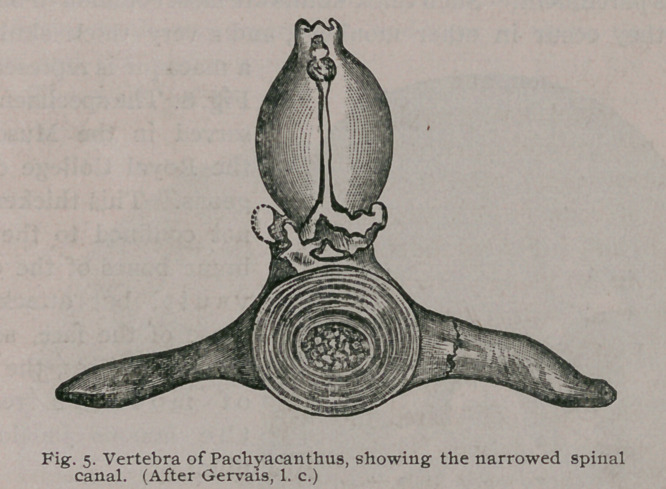


**Fig. 6. f6:**
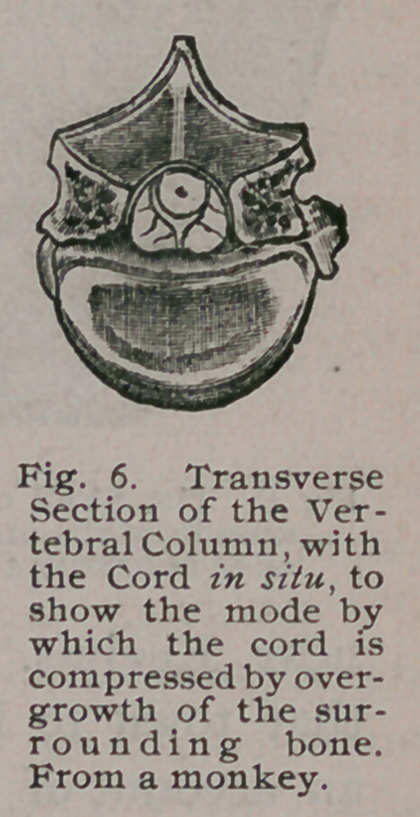


**Fig. 7. f7:**
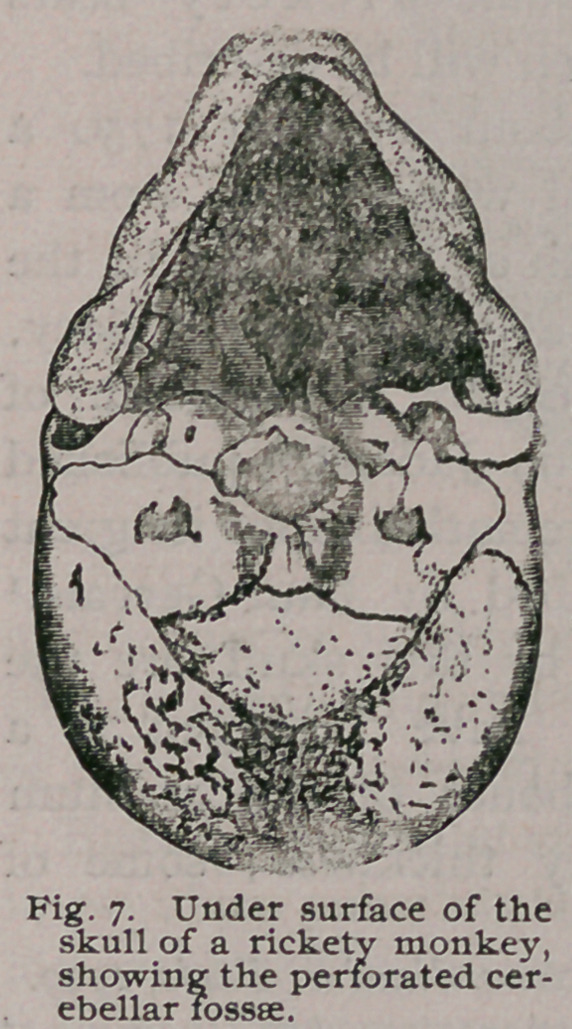


**Fig. 8. f8:**
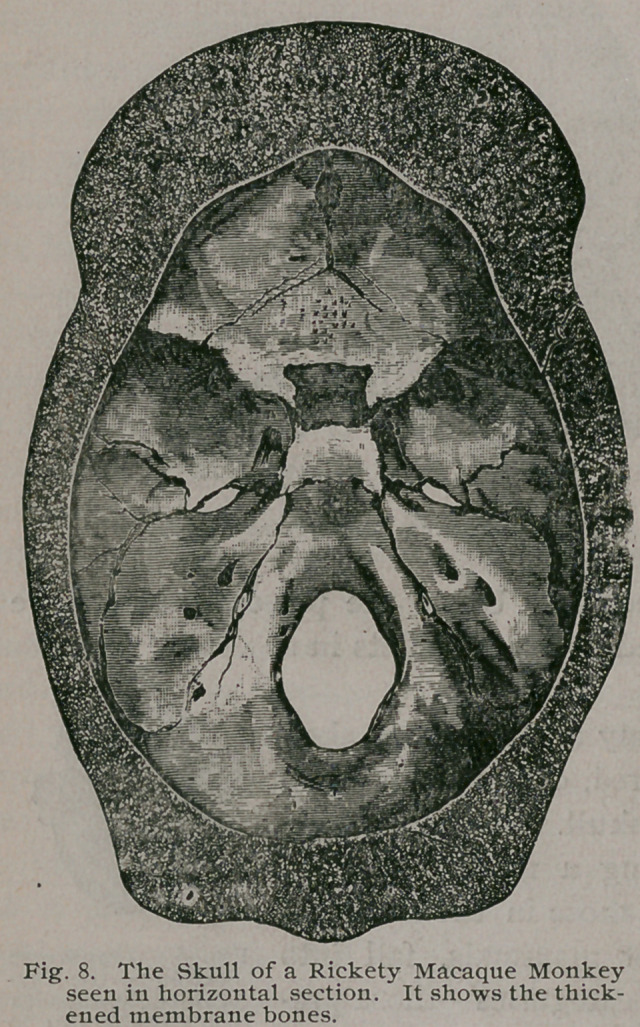


**Fig. 9. f9:**
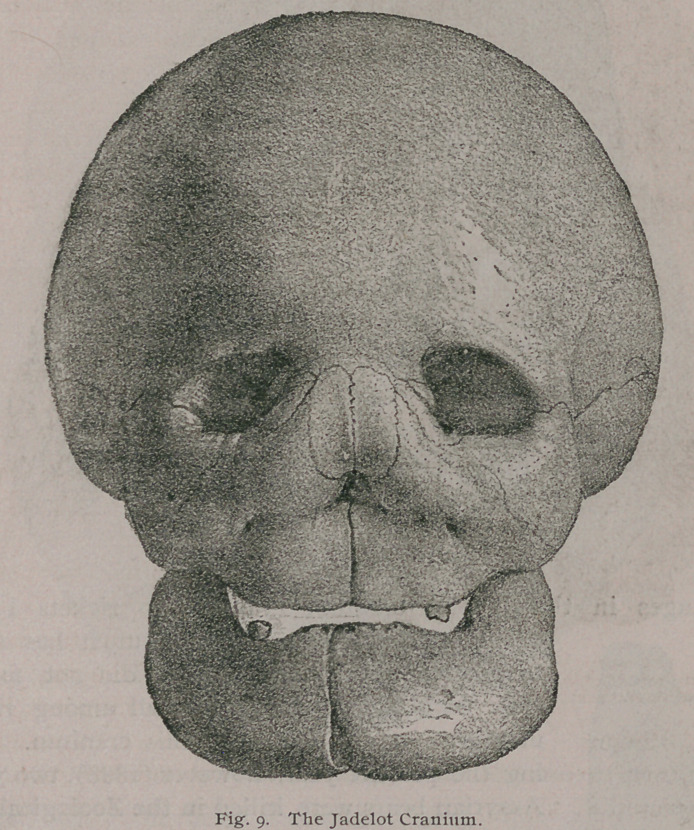


**Fig. 10. f10:**
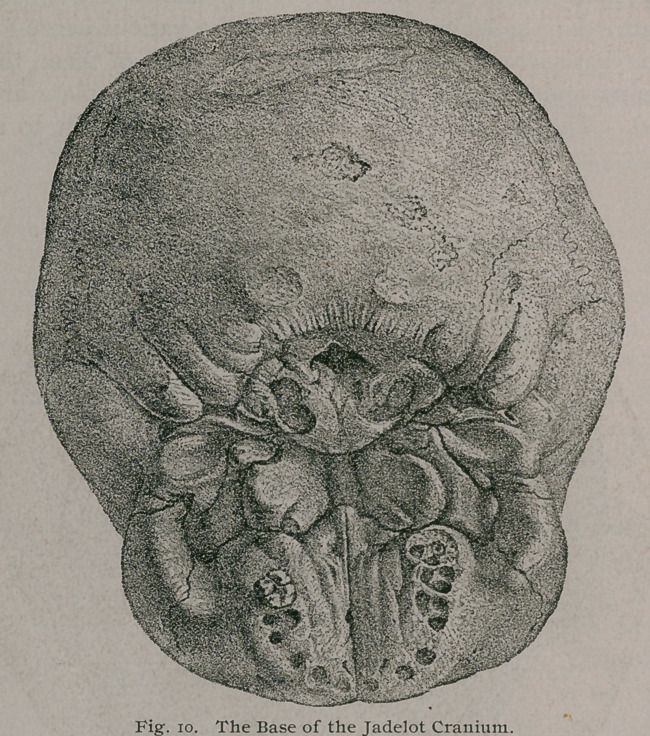


**Fig. 10a. f11:**
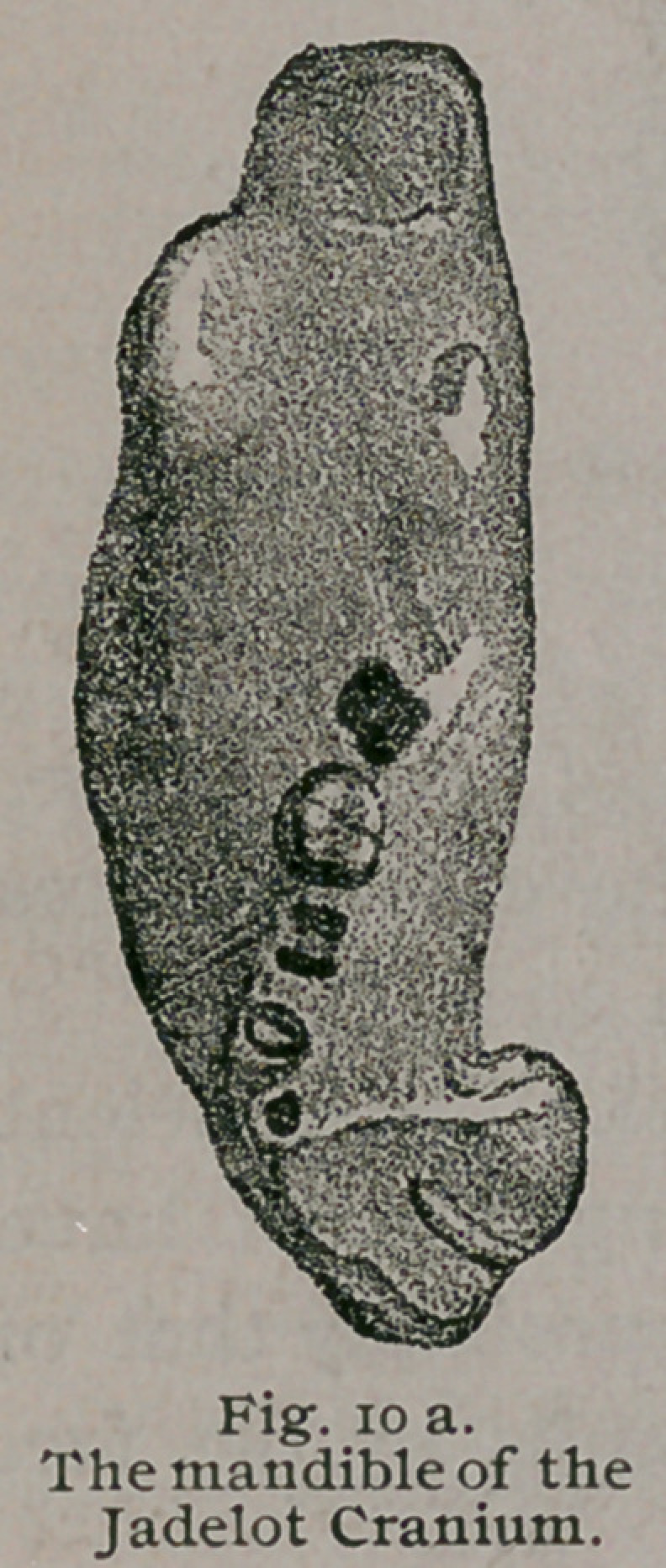


**Fig. 11. f12:**
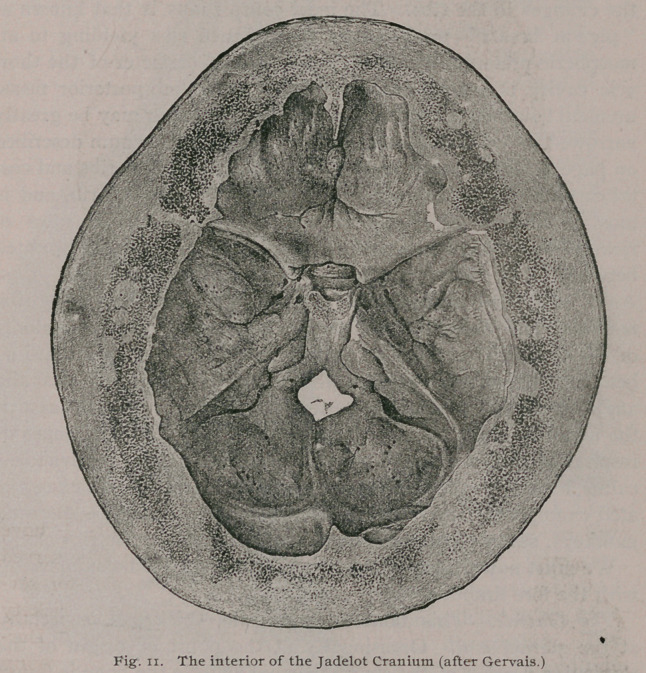


**Fig. 12. f13:**
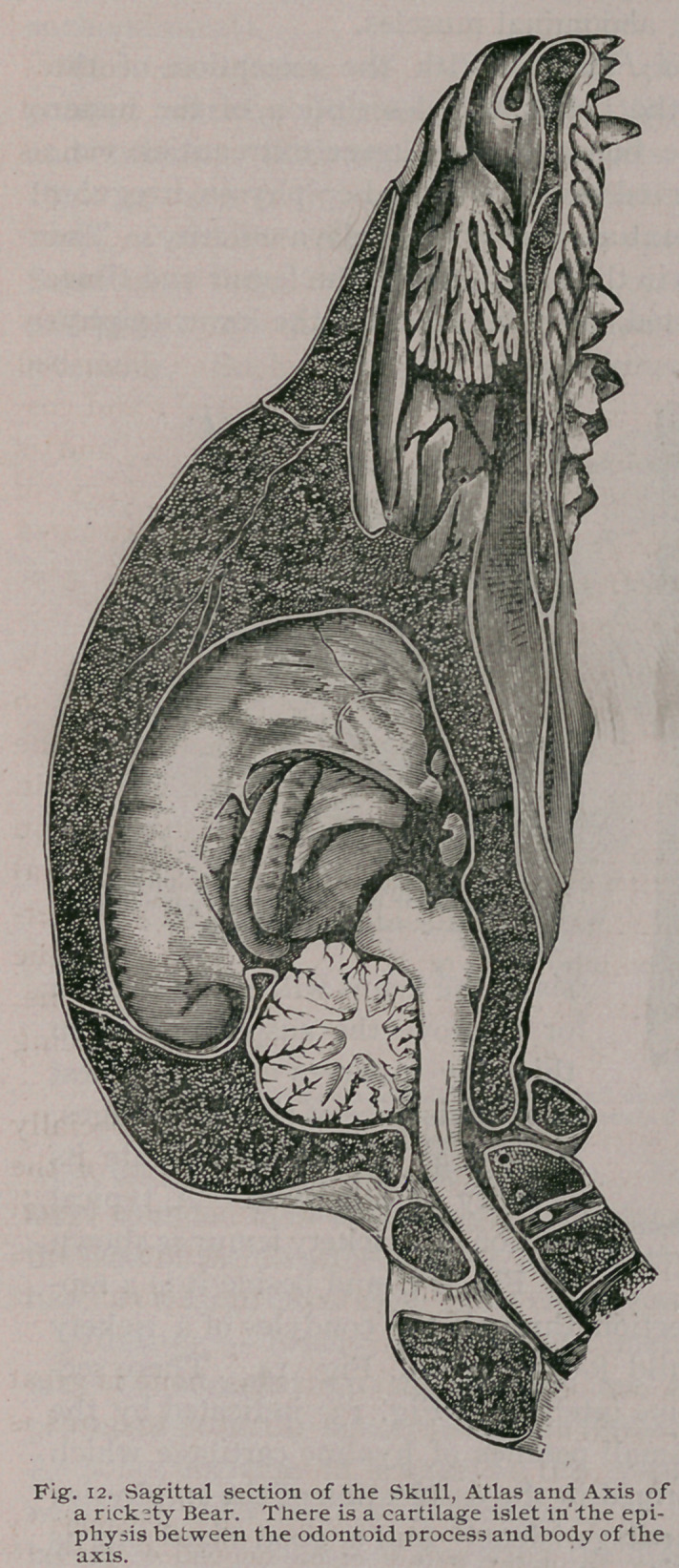


**Fig. 13. f14:**
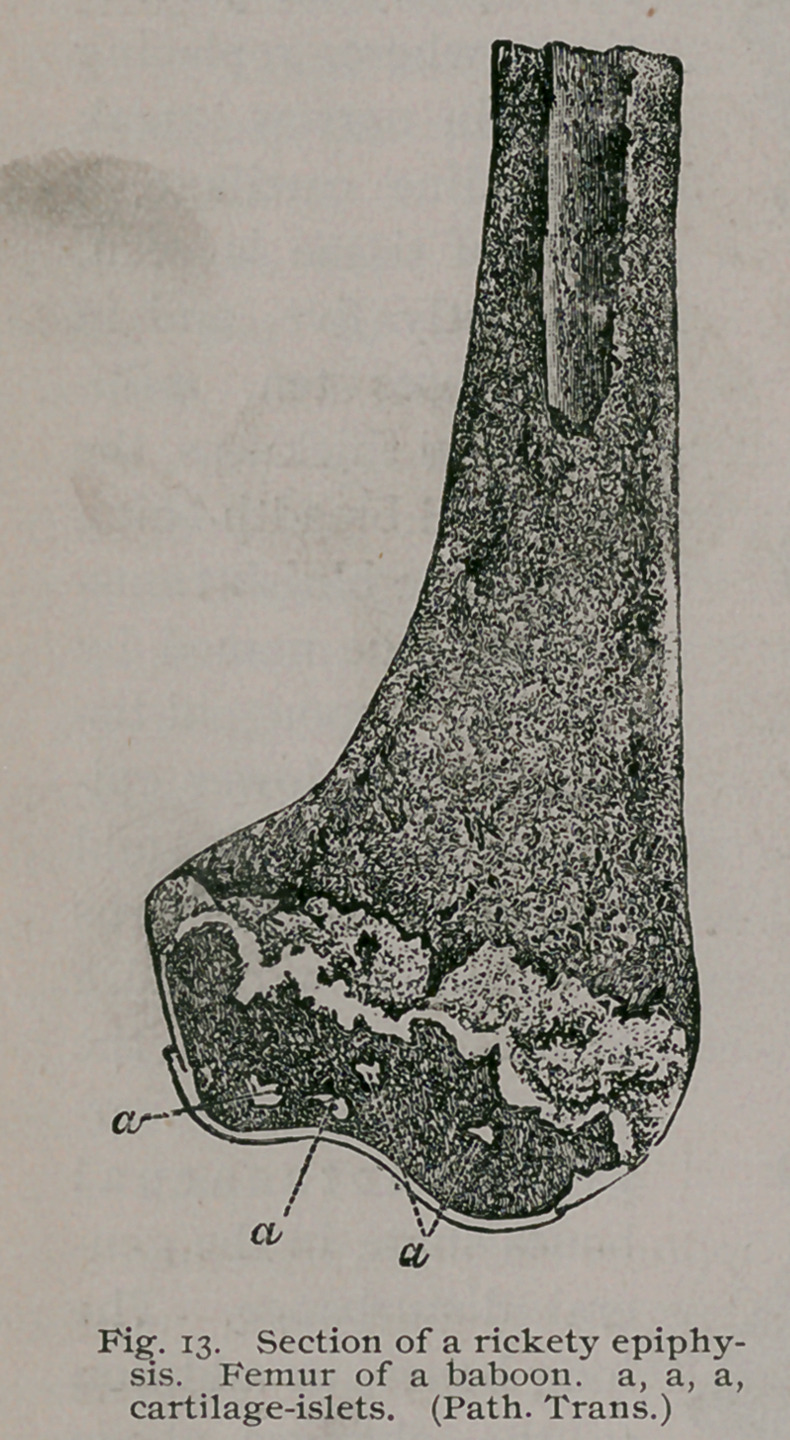


**Fig. 14. f15:**
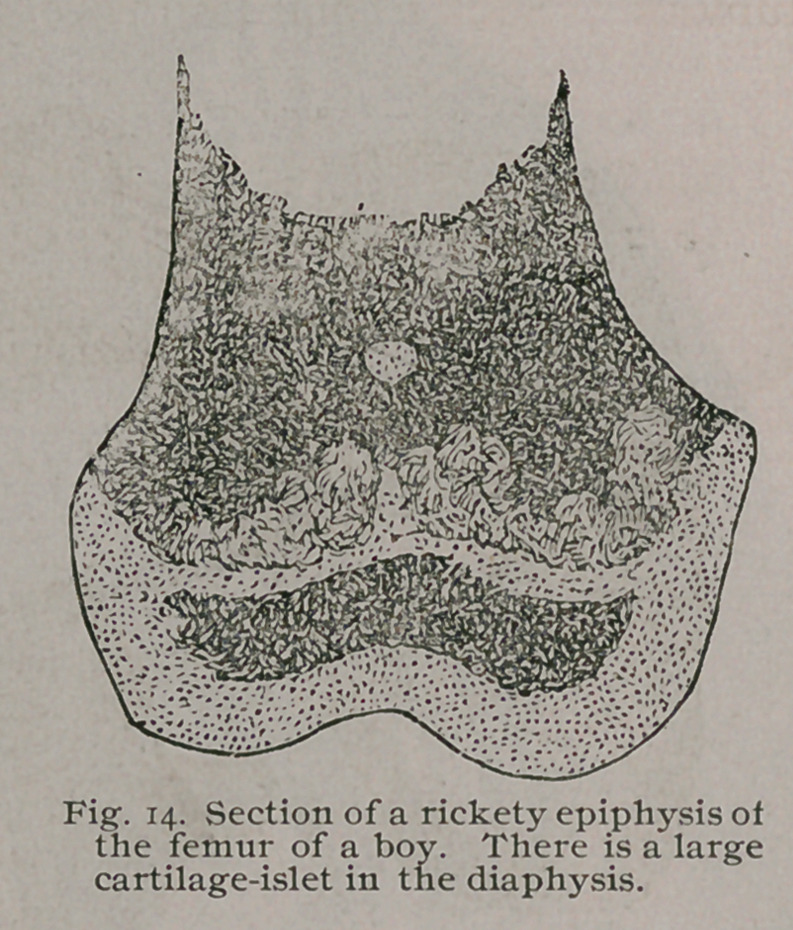


**Fig. 15. f16:**
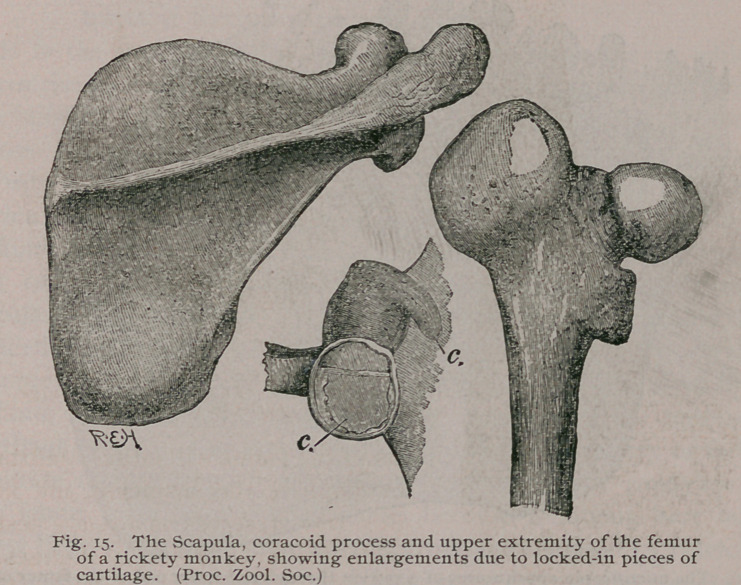


**Fig. 16. f17:**
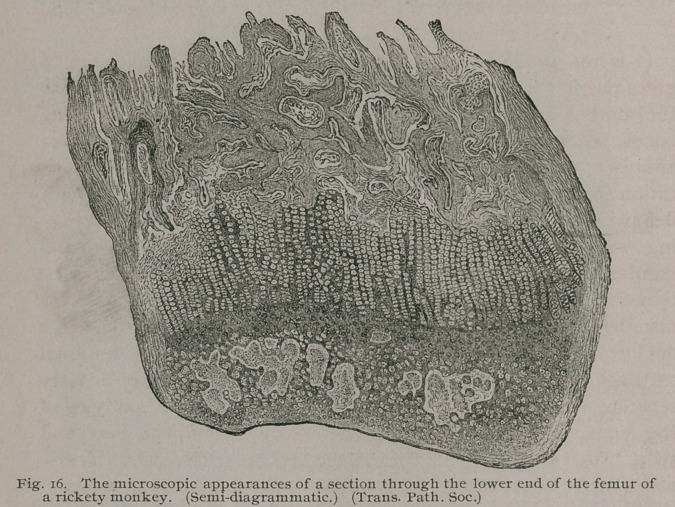


**Fig. 17. f18:**
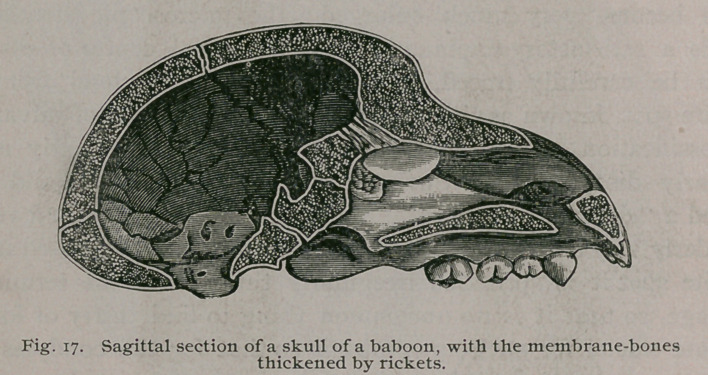


**Fig. 18. f19:**
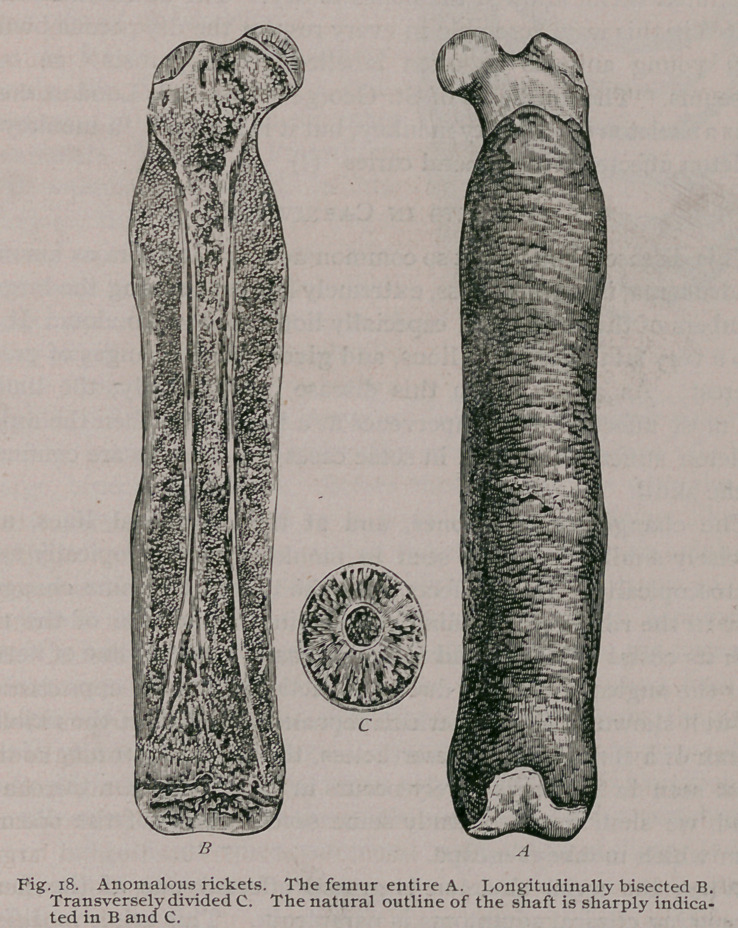


**Fig. 19. f20:**
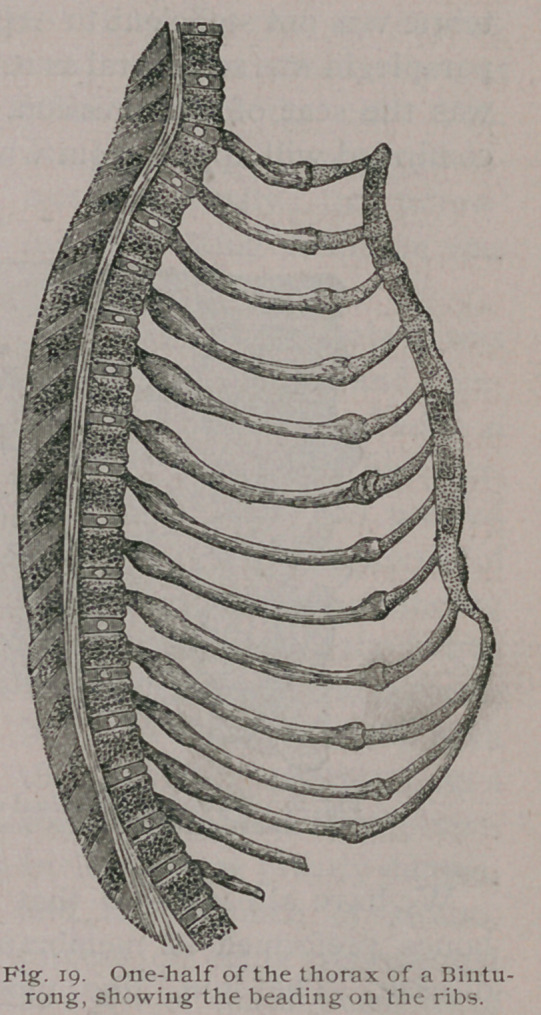


**Fig. 20. f21:**
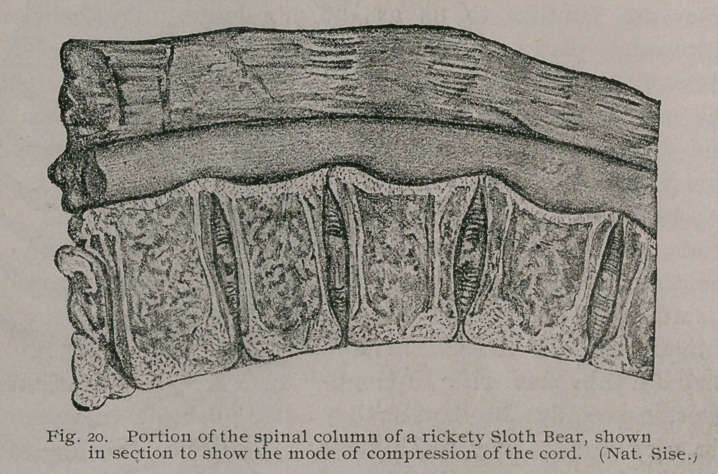


**Fig. 21. f22:**
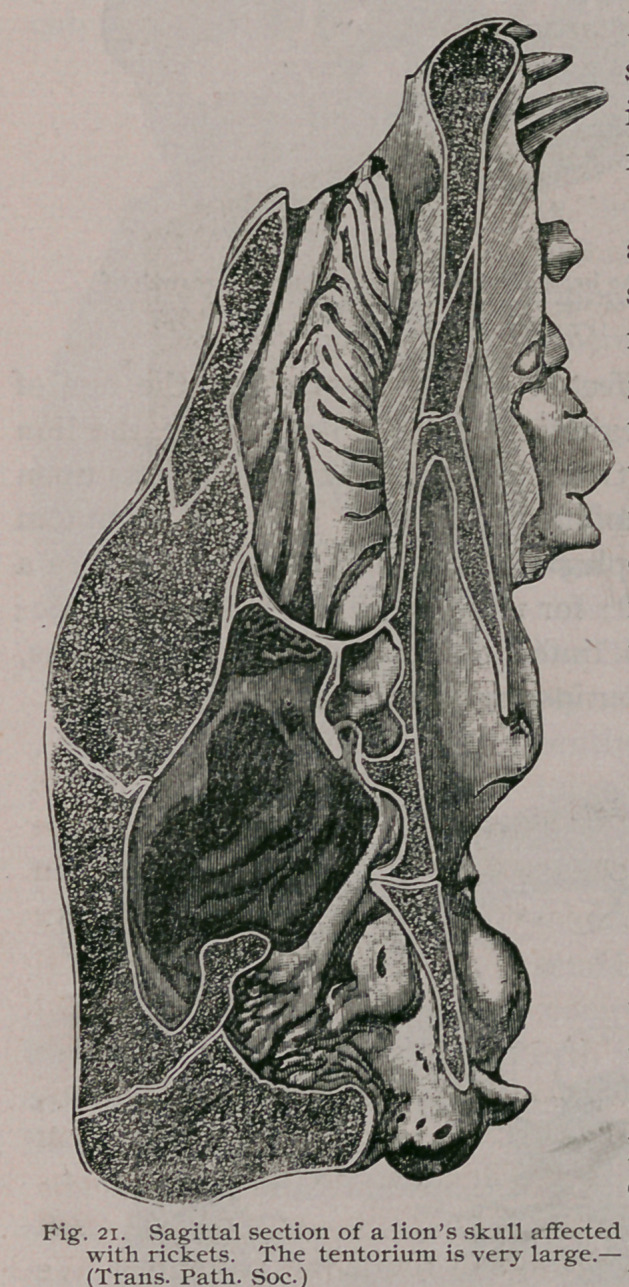


**Fig. 22. f23:**
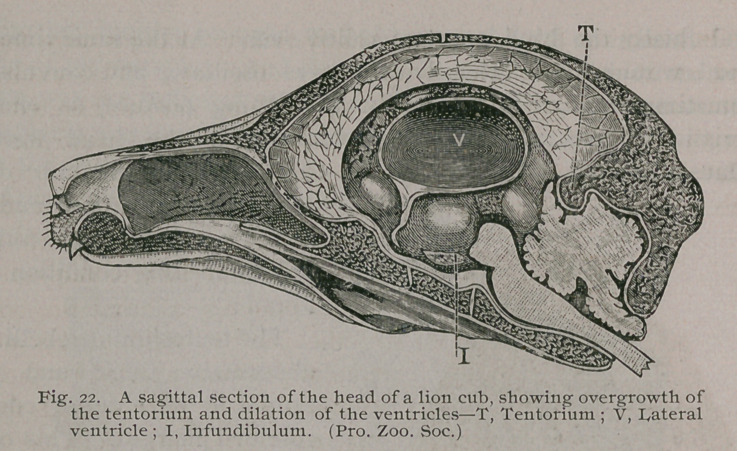


**Fig. 23. f24:**
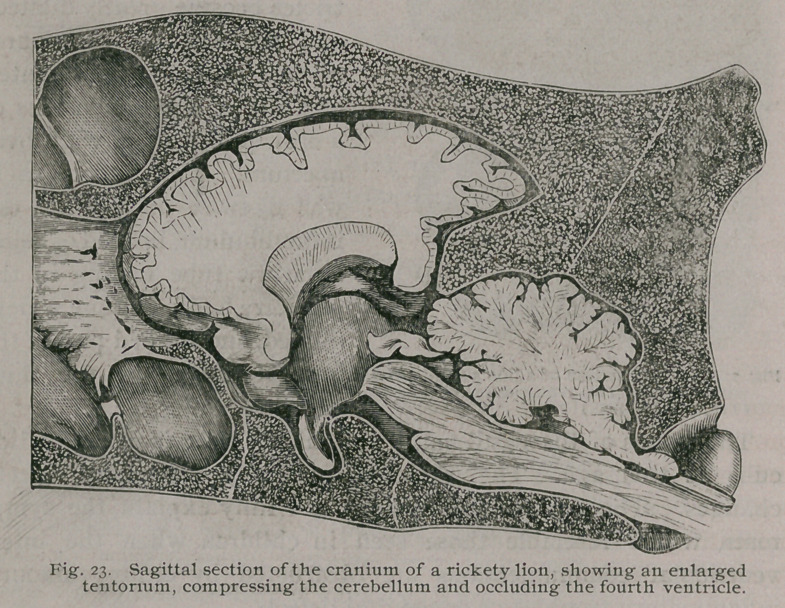


**Fig. 24. f25:**
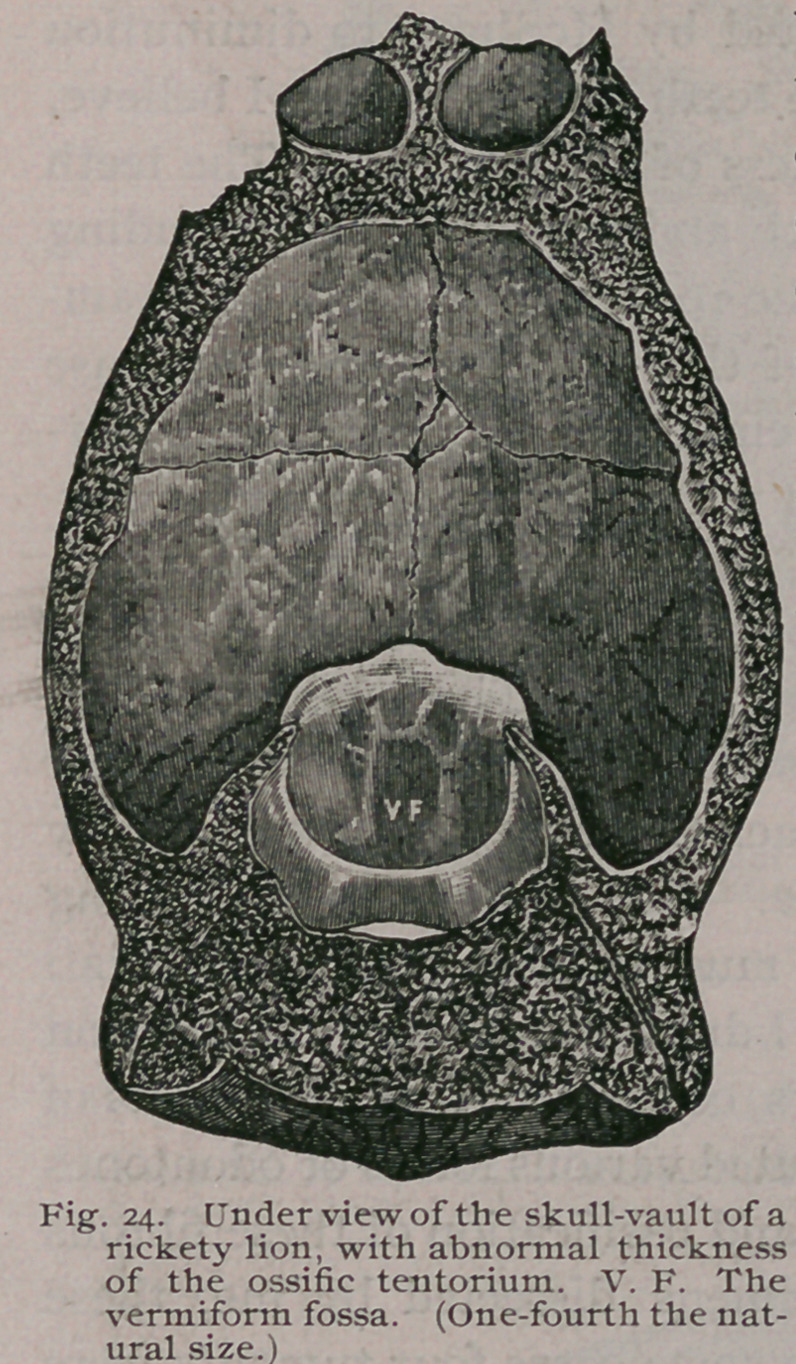


**Fig. 25. f26:**
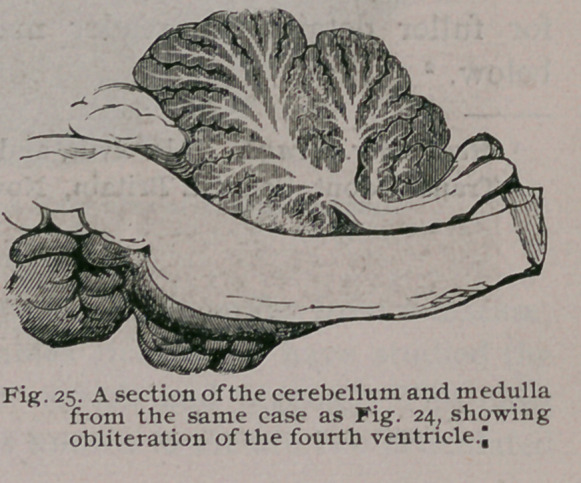


**Fig. 26. f27:**
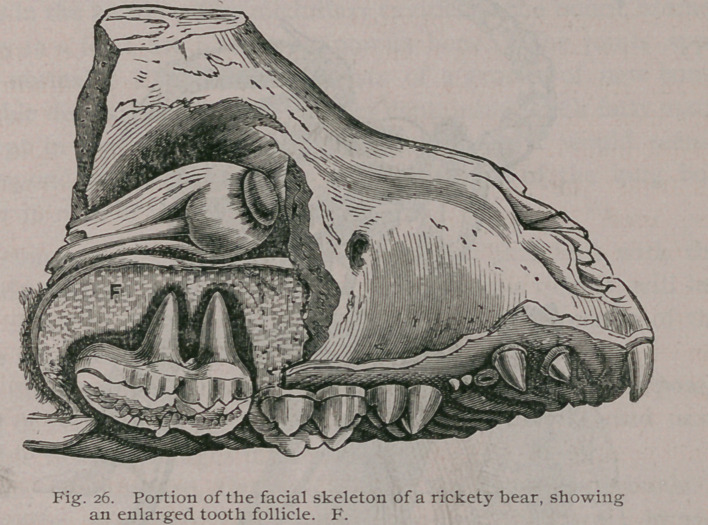


**Fig. 27. f28:**
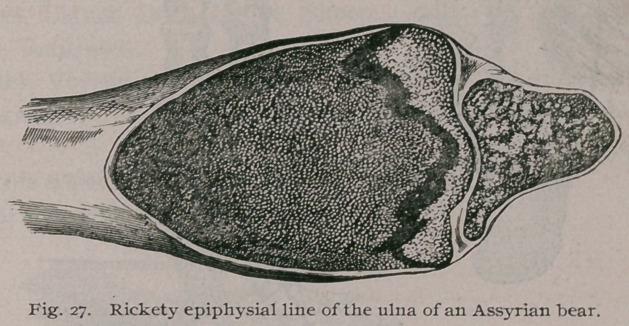


**Fig. 28. f29:**